# Psychopathological Burden in Allergic Contact Dermatitis: Results From a Case–Control Study Using the SCL‐90‐R

**DOI:** 10.1111/cod.70120

**Published:** 2026-02-18

**Authors:** Francisco José Navarro‐Triviño, Álvaro Prados‐Carmona, Ricardo Ruiz‐Villaverde, María Isabel Peralta‐Ramírez

**Affiliations:** ^1^ University of Granada Granada Spain; ^2^ Instituto Biosanitario de Granada, Ibs Granada Spain; ^3^ Department of Dermatology University Hospital San Cecilio Granada Spain; ^4^ Department of Contact Eczema and Immunoallergic Diseases Dermatology, University Hospital San Cecilio Granada Spain; ^5^ Mind, Brain and Behavior Research Center (CIMCYC) University of Granada Granada Spain; ^6^ Department of Personality, Assessment and Psychological Treatment, Faculty of Psychology University of Granada Granada Spain

**Keywords:** allergic contact dermatitis, case–control study, chronic dermatological conditions, mental health impact, psychodermatology, psychopathological symptoms, SCL‐90‐R questionnaire

## Abstract

**Background:**

Allergic Contact Dermatitis (ACD) is a chronic inflammatory skin disease that may be accompanied by psychological distress. Previous studies have suggested an association between ACD and various psychopathological symptoms, which can negatively affect patients' well‐being.

**Objectives:**

To evaluate the psychopathological symptom burden in patients with ACD using the Symptom Checklist‐90‐Revised (SCL‐90‐R) and to explore associations with clinical and sociodemographic variables.

**Patients/Materials/Methods:**

This cross‐sectional case–control study included 450 participants (225 patients with ACD and 225 controls). Group comparisons and association analyses were performed to examine differences between patients and controls and to explore relationships with clinical and sociodemographic characteristics.

**Results:**

Patients with ACD scored significantly higher than controls across all SCL‐90‐R dimensions. The highest proportions of patients scoring above the 70th percentile were observed for phobic anxiety (64.4%), somatization (42.2%) and anxiety (36.9%). Female sex, lower educational level and single marital status were independently associated with higher psychopathological scores. In addition, disease severity was associated with interpersonal sensitivity and psychoticism, while self‐perceived disease severity was associated with somatization and anxiety.

**Conclusions:**

Patients with ACD exhibit a significantly higher burden of psychopathological symptoms compared with controls. This finding highlights the relevance of psychological distress as a common feature of ACD and supports the inclusion of mental health considerations in the clinical assessment of these patients.

## Graphical abstract

This case–control study shows that patients with allergic contact dermatitis have a higher burden of psychopathological symptoms than controls, particularly in phobic anxiety and somatization, highlighting the frequent coexistence of psychological distress in allergic contact dermatitis.

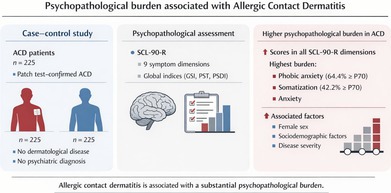

## 1 Introduction

Allergic contact dermatitis (ACD) is a biphasic, immune‐mediated inflammatory reaction triggered by sensitization to a specific allergen [1]. It is a highly prevalent condition in the general population, with a global prevalence estimated at 20.1% according to a recent meta‐analysis [2]. Its clinical manifestations include pruritic eczematous lesions with a heterogeneous presentation ranging from mild erythema to severe vesiculation and crusting. Additionally, non‐eczematous presentations of ACD have been documented [3], potentially complicating and delaying accurate diagnosis.

While the psychological impact of other chronic dermatoses, such as psoriasis [4] and atopic dermatitis (AD) [5] has been well established, particularly their strong association with anxiety and depression [6, 7]. The psychopathological impact of ACD has received less attention. A recent meta‐analysis documented high prevalences of stress (39.4%), depression (27.2%) and anxiety (28.8%) in patients with chronic dermatoses [8]. Similarly, hand eczema has been observed to significantly affect quality of life (QoL), being associated with elevated anxiety levels and an increased risk of psychological disorders [9]. These findings highlight the complex relationship between skin conditions and mental health.

Addressing the psychological needs of ACD patients is essential, as psychiatric comorbidity, closely linked to disease severity [10], can significantly compromise QoL, regardless of patch testing outcomes [11]. For instance, patients with hand eczema exhibit higher rates of depression (20.3% vs. 15.4%) and anxiety (10.6% vs. 7.8%) compared to those without eczema, with a significantly increased odds ratio for psychiatric morbidity (OR 1.40, 95% CI: 1.10–1.78) [12].

Despite these insights, the psychopathological profile of patients with ACD remains insufficiently characterised in the literature. This study systematically evaluates the psychopathological burden of ACD, using an integrative approach that incorporates clinical and sociodemographic data. This research aims to characterise the presence and severity of psychopathological symptoms in ACD patients versus healthy controls and quantify the prevalence of clinically relevant psychopathological scores using a validated multidimensional tool (SCL‐90‐R). Secondary objectives include exploring the influence of sociodemographic factors and examining potential correlations with clinical variables such as lesion severity, disease duration and history of prior emergency department visits.

## 2 Materials or Methods

### 2.1 Study Design and Setting

This case–control study was conducted at the Immunoallergy Unit of the Dermatology Department at a tertiary hospital in Spain between January 2021 and December 2023. Patients with ACD were consecutively recruited during routine outpatient consultations.

### 2.2 Participants

#### 2.2.1 ACD Patients

Inclusion criteria for the ACD group were age ≥ 18 years, sufficient Spanish literacy and a confirmed diagnosis of ACD, defined by at least one positive patch test to a clinically relevant allergen and a compatible clinical pattern.

Exclusion criteria included intellectual disability, any previously diagnosed psychiatric disorder, current or past psychopharmacological treatment and the presence of other chronic dermatological diseases unrelated to ACD, including AD, psoriasis, lichen planus, drug eruptions, or other inflammatory dermatoses.

#### 2.2.2 Control Group

The control group was recruited using a non‐probability convenience sampling strategy, primarily among companions or relatives of dermatology outpatients, to enhance sociodemographic comparability and minimise the influence of uncontrolled contextual variables.

Controls met the same general eligibility criteria as patients and had no current or past dermatological disease, no history of ACD or positive patch testing and no known psychiatric diagnosis or history of psychopharmacological treatment. Psychiatric eligibility was assessed through a brief screening interview and review of self‐reported medical and medication history.

Controls were not systematically screened for subclinical psychological distress; however, individuals with a known psychiatric diagnosis or ongoing psychopharmacological treatment were excluded. This limitation was considered when interpreting the results.

### 2.3 Sample Size Calculation

The total study population comprised 450 participants, including 225 patients with ACD and 225 controls. Sample size estimation was based on previous studies assessing the psychological impact of chronic inflammatory dermatoses, assuming a significance level (*α*) of 0.05 and a statistical power (1 − *β*) of 0.80 to detect medium‐sized effects (Cohen's *d* = 0.5). This approach ensured adequate power for between‐group comparisons while minimising the risk of type I and type II errors.

### 2.4 Ethical Considerations

The study was approved by the Institutional Medical Research Ethics Committee (DERM_HUSC_‐2021) and conducted in accordance with the principles of the Declaration of Helsinki. All participants received detailed information regarding the study objectives, confidentiality and the voluntary nature of participation. Written informed consent was obtained from all participants before inclusion.

### 2.5 Instruments

Sociodemographic and clinical variables were obtained during a structured medical interview. Recorded variables included age, sex, educational level, marital status, occupational status and disease duration. Disease duration was recorded in months based on the patient‐reported time since symptom onset. For the control group, only sociodemographic data and mental health‐related variables required to confirm eligibility were documented.

The diagnosis of ACD was confirmed through patch testing using the national standard series and extended/specific allergen series provided by Chemotechnique Diagnostics (Vellinge, Sweden), tailored to each case. Patch test results were interpreted following the recommendations of the European Society of Contact Dermatitis (ESCD) [13]. The clinical relevance of positive reactions was assessed based on the source of exposure and the plausibility of a causal relationship between allergen exposure and the patient's dermatitis.

### 2.6 Severity Variables

Disease severity at the initial visit was assessed using the medical Investigator Global Assessment (mIGA) scale [14]. This scale categorises severity levels as follows: 0 (no lesions), 1 (very mild lesions with barely perceptible redness or scaling), 2 (mild lesions with visible but limited redness or scaling), 3 (moderate lesions with clearly visible redness and scaling across multiple areas) and 4 (severe lesions with extensive redness, significant scaling and in some cases, vesicles or erosions). The mIGA scale has demonstrated reliability indices of 0.516–0.778 and validity indices of 0.497–0.893.

Although originally validated for AD, the mIGA scale has been previously applied in the context of ACD to assess disease severity [15], supporting its use as a practical tool in this context. Additionally, evidence suggests that ACD and AD share similar clinical and immunological features, further justifying the application of this scale in ACD research [16].

Patients' self‐perception of disease severity and concern about lesions were assessed using numerical rating scales (NRS) ranging from 0 (no concern) to 10 (worst imaginable condition).

### 2.7 Assessment of Psychopathological Symptoms

Psychopathological symptoms were assessed using the Symptom Checklist‐90‐Revised (SCL‐90‐R), a widely used self‐report instrument designed to measure psychological distress across nine symptom dimensions: somatization, obsessive‐compulsive traits, interpersonal sensitivity, depression, anxiety, hostility, phobic anxiety, paranoid ideation and psychoticism [17]. Each item is rated on a 5‐point Likert scale (0–4), reflecting the degree of distress experienced during the previous week. Raw scores for each subscale were calculated as the mean of the corresponding item scores.

In addition to the nine symptom dimensions, three global indices were derived: the Global Severity Index (GSI), reflecting overall psychological distress by integrating symptom intensity and number; the Positive Symptom Total (PST), representing the number of items with a score greater than zero and providing an estimate of symptom breadth and the Positive Symptom Distress Index (PSDI), reflecting the average intensity of reported symptoms, calculated as the sum of item scores divided by the PST.

Raw scores were subsequently transformed into percentile scores based on normative reference data from the SCL‐90‐R manual, allowing comparison with the general population. According to the manual, percentile values equal to or above the 70th percentile, approximately corresponding to scores two standard deviations above the normative mean, are commonly interpreted as indicative of clinically relevant psychological symptomatology. In the present study, the 70th percentile was used as a descriptive threshold to identify participants with a relatively high burden of psychopathological symptoms.

The Spanish‐validated version of the SCL‐90‐R was used, as it has demonstrated robust psychometric properties in Spanish‐speaking populations. Reported internal consistency coefficients (Cronbach's alpha) range from 0.69 to 0.95, supporting its reliability and suitability for the assessment of psychopathological symptoms in clinical research [18].

### 2.8 Statistical Analysis

Statistical analyses were performed to evaluate differences and associations between sociodemographic, clinical and psychological variables. Categorical variables were compared between the ACD and control groups using chi‐square tests, while differences in continuous outcomes were primarily assessed using Student's *t*‐tests.

Although individual SCL‐90‐R items are rated on ordinal Likert‐type scales, subscale and global index scores represent continuous composite measures, calculated as the mean of the corresponding item scores, as recommended in the SCL‐90‐R manual. Normality of score distributions was assessed using the Shapiro–Wilk test. When deviations from normality were observed, non‐parametric Mann–Whitney *U* tests were conducted as sensitivity analyses to confirm the robustness of between‐group comparisons.

Within the ACD group, correlation analyses and group comparisons were performed to explore associations between psychopathological symptom scores and relevant clinical and sociodemographic variables, including age, educational level, marital status, disease duration, history of emergency visits and anatomical distribution of lesions.

To identify variables independently associated with psychopathological symptom burden, multiple linear regression analyses were conducted using continuous SCL‐90‐R subscale and global index scores as dependent variables, with sociodemographic and clinical variables included as independent predictors. Regression results were interpreted with appropriate caution, focusing on the direction and relative magnitude of associations.

A two‐sided significance level of *p* < 0.05 was applied for all analyses, and results are presented with 95% confidence intervals where appropriate. Statistical analyses were performed using SPSS for Windows, version 27.0 (IBM Corp., Armonk, NY).

## 3 Results

### 3.1 Sample Description

The study included a total of 450 participants, comprising 225 patients with ACD and 225 controls. Both groups were well‐matched in key sociodemographic and educational variables, with no statistically significant differences observed between them (Table 1).

**TABLE 1:** Sociodemographic and clinical variables in patients with allergic contact dermatitis and the control group.

Sociodemographic variables	ACD group (*n* = 225), *M* (SD)/*n* (%)	Control group (*n* = 225), *M* (SD)/*n* (%)	*p* (*t/χ* ^2^)
Age	33.01 (4.561)	33.56 (4.327)	0.468
Sex			0.611
Women	152 (67.6%)	157 (69.8%)	
Men	73 (32.4%)	68 (30.2%)	
Marital status			0.953
Single	76 (33.8%)	80 (35.6%)	
Married	117 (52%)	111 (49.3%)	
Divorced	23 (10.2%)	24 (10.7%)	
Widowed	9 (4%)	10 (4.4%)	
Educational level			0.592
Basic education	16 (7.1%)	18 (7.8%)	
Primary education	19 (8.4%)	19 (8.4%)	
Secondary education	38 (16.9%)	32 (14.2%)	
High school	39 (17.3%)	41 (18.2%)	
University studies	113 (50.2%)	115 (51.1%)	
Employed	138 (61.33%)	124 (55.11%)	0.214

In the ACD group, the mean disease duration was 68.26 months (SD = 110.0). Over the past 12 months, 40.89% of patients have sought emergency care due to ACD, with a mean of 1.61 visits (SD = 2.81). The average mIGA score for disease severity was 2.31 (SD = 1.06).

Regarding patient‐reported burden, the mean NRS scores were 6.29 (SD = 2.90) for functional limitation and 7.18 (SD = 2.70) for concern about the condition. Additionally, patients' self‐perceived severity had a mean score of 5.37 (SD = 3.02).

### 3.2 Comparison of Psychopathological Symptoms Between ACD Patients and Controls

Comparison between patients with ACD and the control group showed significantly higher mean scores in the ACD group across all SCL‐90‐R symptom dimensions, including somatization, obsessive‐compulsive symptoms, interpersonal sensitivity, depression, anxiety, hostility, phobic anxiety, paranoid ideation and psychoticism (Table 2). These differences remained consistent in non‐parametric sensitivity analyses, confirming the robustness of the between‐group comparisons.

**TABLE 2:** Comparison of SCL‐90‐R dimensions and indices between allergic contact dermatitis group and control group (mean score ± SD).

SCL‐90R subscale	ACD group, *M* (SD)	Control group, *M* (SD)	*p*
Somatization	1.08 (0.79)	0.31 ± 0.19	0.001**
Obsessive‐Compulsive	1.31 ± 0.81	0.33 ± 0.22	0.001**
Interpersonal Sensitivity	0.90 ± 0.74	0.34 ± 0.22	0.001**
Depression	1.13 ± 0.82	0.31 ± 0.18	0.001**
Anxiety	0.98 ± 0.74	0.32 ± 0.20	0.001**
Hostility	0.65 ± 0.60	0.31 ± 0.28	0.001**
Phobic Anxiety	0.86 ± 0.63	0.33 ± 0.24	0.001**
Paranoid Ideation	0.76 ± 0.74	0.35 ± 0.28	0.001**
Psychoticism	0.56 ± 0.59	0.33 ± 0.20	0.001**
Global Severity Index	0.94 ± 0.64	0.32 ± 0.07	0.001**
Positive Symptom Total	43.25 (21.23)	23.27 ± 4.10	0.001**
Positive Symptom Distress Index	1.80 ± 0.51	2.16 ± 0.13	0.001**

** *p* < 0.02.

**FIGURE 1:**
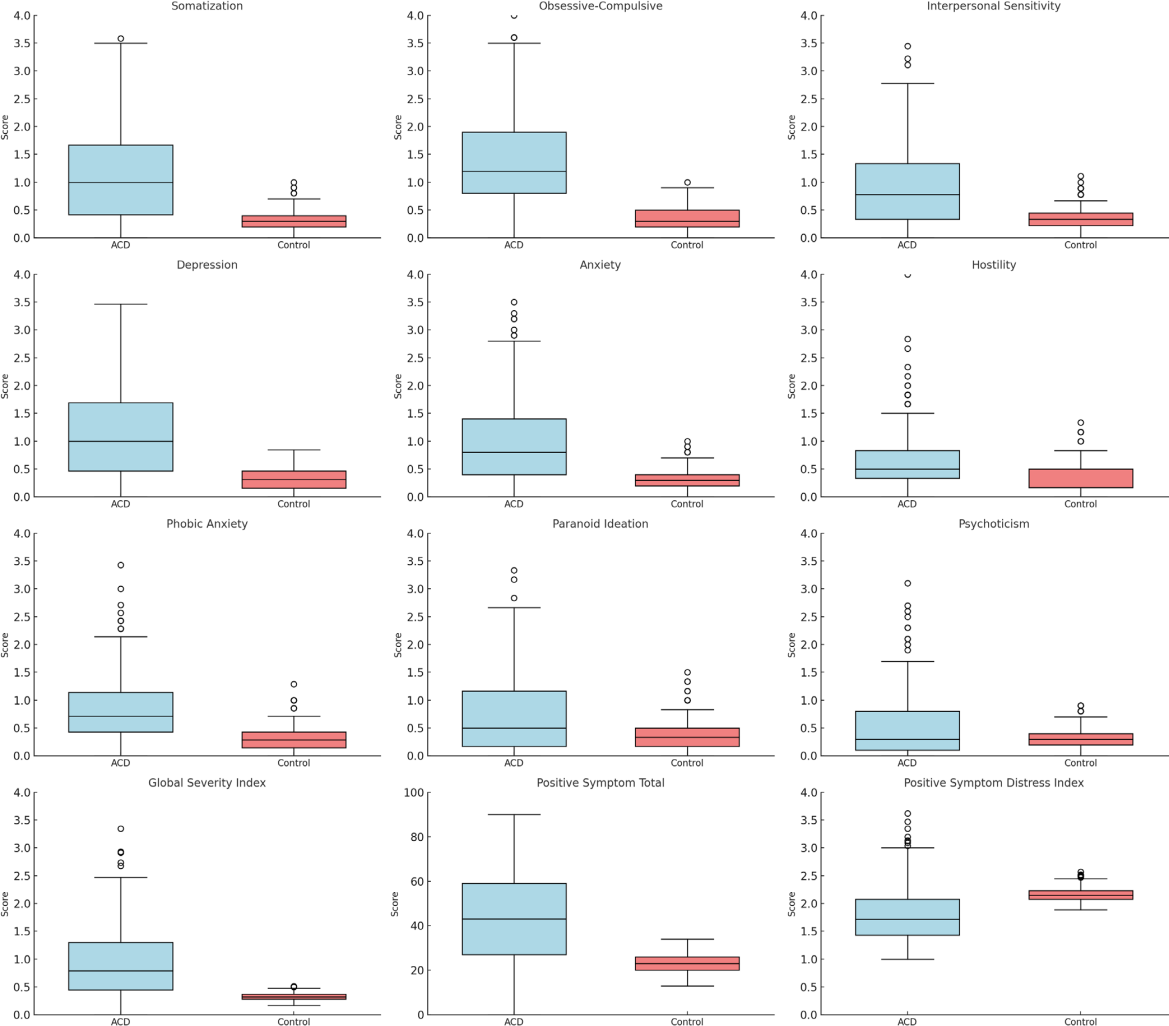
Boxplots of SCL‐90‐R psychopathological dimensions and indexes comparing allergic contact dermatitis patients and control group.

In addition, global psychopathological indices, namely GSI, PST and PSDI, were significantly higher in ACD patients than in controls (Figure 1).

**FIGURE 2:**
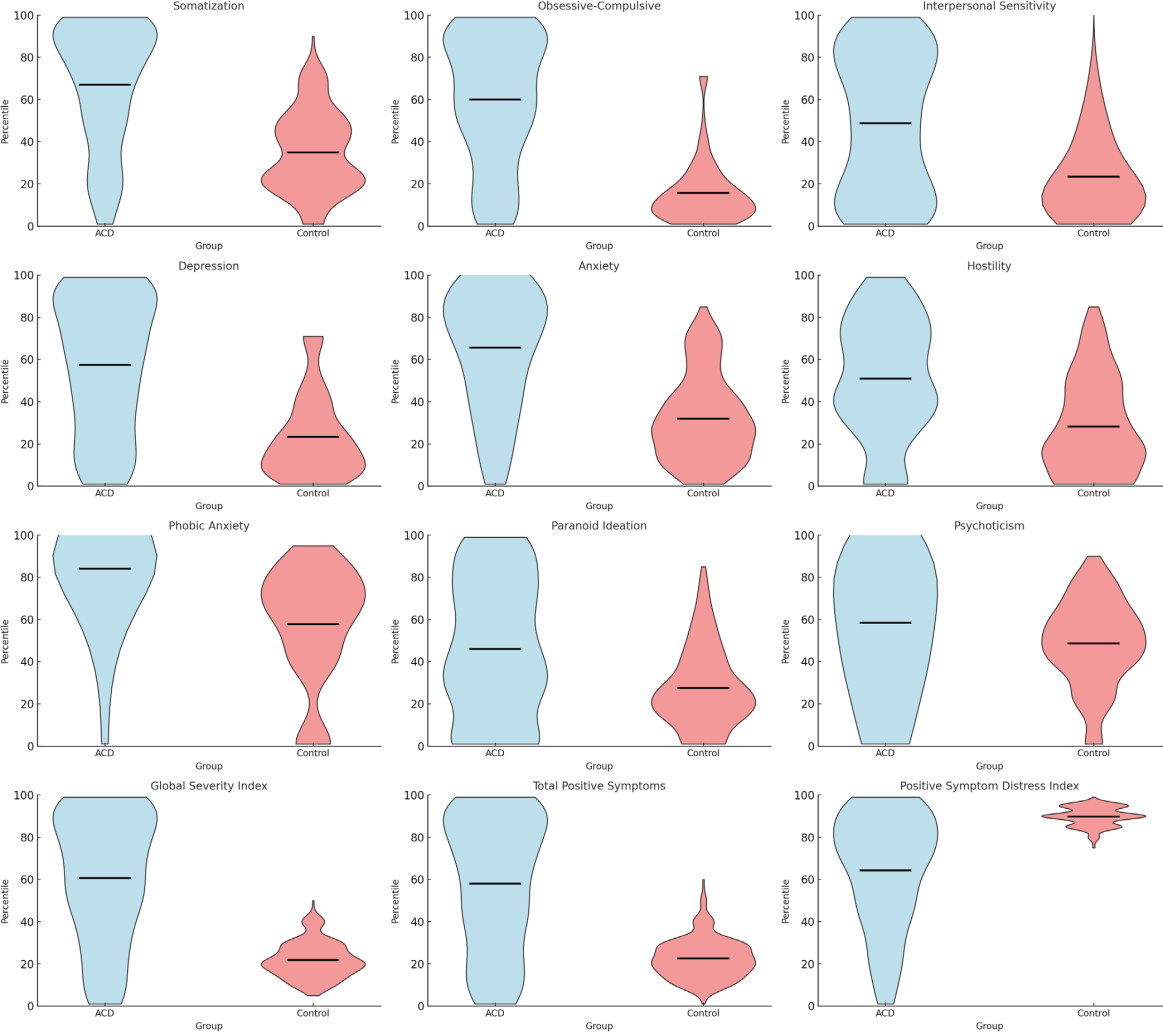
Violin plots of percentile distributions for SCL‐90‐R dimensions and indexes in allergic contact dermatitis patients and control group. The central black line within each violin represents the median percentile value.

To further characterise the distribution of psychopathological symptoms, a percentile‐based comparative analysis using the 70th percentile threshold was performed (Figure 2). A notably higher proportion of ACD patients scored above the 70th percentile, reflecting an increased burden of psychopathological symptoms compared with controls. The highest proportions were observed for phobic anxiety (64.44% of patients above the 70th percentile), followed by somatization (42.22%), anxiety (36.89%), obsessive‐compulsive symptoms (31.56%) and depression (31.11%). Additional dimensions with elevated scores included psychoticism (26.22%), interpersonal sensitivity (23.11%), paranoid ideation (17.78%) and hostility (15.11%) (Table 3). Among the SCL‐90‐R dimensions, phobic anxiety and somatization showed the highest proportions of patients scoring above the 70th percentile in the ACD group compared with controls.

**TABLE 3:** Distribution of study participants in allergic contact dermatitis and control groups above the 70th percentile across SCL‐90‐R dimensions.

SCL‐90 subscale	ACD > 70 (%) (*n*)	Control > 70 (%) (*n*)
Somatization	53.33% (120)	6.22% (14)
Obsessive‐Compulsive	40.44% (91)	3.56% (8)
Interpersonal Sensitivity	32.00% (72)	2.67% (6)
Depression	38.22% (86)	7.56% (17)
Anxiety	47.56% (107)	9.33% (21)
Hostility	26.22% (59)	4.44% (10)
Phobic Anxiety	78.67% (177)	28.89% (65)
Paranoid Ideation	27.11% (61)	2.22% (5)
Psychoticism	35.56% (80)	11.11% (25)

### 3.3 Associated Variables and Predictors of Psychopathological Symptoms in ACD Patients

To explore sociodemographic and disease‐related variables associated with psychopathological symptoms in ACD patients, several analyses were conducted.

#### 3.3.1 Sociodemographic Variables

Correlation analyses showed statistically significant associations between age and several psychopathological dimensions. Age was positively correlated with phobic anxiety (*r* = 0.390, *p* < 0.01), psychoticism (*r* = 0.114, *p* < 0.05) and the GSI (*r* = 0.122, *p* < 0.05).

Statistically significant sex‐related differences were observed across multiple SCL‐90‐R dimensions. Women showed higher scores than men in somatization (*p* = 0.006), interpersonal sensitivity (*p* = 0.022), depression (*p* = 0.002), anxiety (*p* = 0.036), hostility (*p* = 0.017), phobic anxiety (*p* = 0.017), paranoid ideation (*p* = 0.019) and psychoticism (*p* = 0.041). Similarly, global indices, including GSI (*p* = 0.005) and PST (*p* < 0.001), were higher among women.

Marital status was significantly associated with differences in depression (*p* = 0.023), paranoid ideation (*p* = 0.041) and GSI (*p* = 0.031). Specifically, single individuals scored significantly higher in depression (*p* = 0.030) and psychoticism (*p* = 0.041) compared to married or partnered individuals. Additionally, divorced/separated individuals had significantly higher scores in paranoid ideation (*p* = 0.047).

In multiple linear regression analyses, sex was independently associated with somatization (*p* = 0.008) and depression (*p* = 0.004). Interpersonal sensitivity was independently associated with age (*p* = 0.046), gender (*p* = 0.04) and marital status (*p* = 0.014). Marital status was also independently associated with psychoticism (*p* = 0.007) (Table 4).

**TABLE 4:** Multiple linear regression analyses of sociodemographic variables associated with SCL‐90‐R dimensions in patients with allergic contact dermatitis.

Dependent variable	Independent variable	*R* ^2^	Adjusted *R* ^2^	*β*	*t*	*p*
Somatization	Sex	0.054	0.032	−0.177	−2.67	0.008**
Interpersonal sensitivity	Age	0.068	0.046	−0.162	−2.01	0.046*
	Sex			−0.14	−2.07	0.04*
	Marital status			0.205	2.47	0.014**
Depression	Sex	0.086	0.065	−0.19	−2.9	0.004**
	Marital status			0.2	2.43	0.016*
Phobic anxiety	Sex	0.054	0.033	−0.153	−2.32	0.021*
Psychoticism	Marital status	0.056	0.034	0.226	2.71	0.007**

*Note: β* values represent standardised regression coefficients. Only variables with statistically significant associations (*p* < 0.05) are shown.

* *p* < 0.05.

** *p* < 0.02.

#### 3.3.2 Disease‐Specific Variables

Regarding disease‐related variables, multiple linear regression with psychoticism was performed for selected SCL‐90‐R dimensions. For phobic anxiety, the regression model was statistically significant (*F*(3, 220) = 2.72, *p* < 0.045), explaining 3.6% of the variance (*R* ^2^ = 0.036; adjusted *R* ^2^ = 0.023). Disease duration was independently associated with phobic anxiety scores (*B* = 0.176, *t* = 0.26, *p* < 0.01), whereas disease severity and the number of emergency department visits were not significantly associated.

For general anxiety, the regression model was also statistically significant (*F*(3, 220) = 4.04, *p* = 0.008), explaining 5.2% of the variance (*R* ^2^ = 0.052; adjusted *R* ^2^ = 0.039). This model was independently associated with anxiety scores (*B* = 0.23, *t* = 3.46, *p* < 0.001), while disease severity and disease duration were not significantly associated.

## 4 Discussion

This study provides comprehensive insights into the psychopathological burden associated with ACD. The primary objective was to evaluate whether individuals with ACD exhibit a higher level of psychopathological symptoms compared to controls. Our results show that ACD patients presented significantly higher scores across all SCL‐90‐R dimensions than controls.

In addition, the percentile‐based analysis demonstrated that a substantial proportion of ACD patients scored at or above the 70th percentile across several symptom dimensions, particularly anxiety, phobic anxiety and somatization, indicating a higher burden of psychopathological symptoms in this population.

The SCL‐90‐R is a widely used instrument for assessment of psychopathological symptoms across a broad range of medical conditions, including dermatological diseases. Previous studies in patients with moderate‐to‐severe psoriasis [19] have consistently reported an increased prevalence of psychopathological symptoms, particularly depressive symptoms, compared with the control population. Similarly, research in other chronic skin conditions, such as chronic spontaneous urticaria, has described a high frequency of psychosomatic symptoms, including anxiety and somatization [20]. Furthermore, patients with lichen simplex chronicus [21] have exhibited significantly higher mean scores on the SCL‐90‐R compared to controls, with notable associations with dissociative experiences [21].

To date, research on ACD has largely focused on its impact on QoL, particularly in occupational settings and in cases involving hand eczema [22, 23]. Hand involvement has been consistently associated with increased levels of anxiety, depressive symptoms and obsessive‐compulsive traits [24], and heightened psychological stress [25].

However, beyond these specific clinical contexts, the broader psychopathological profile associated with ACD has been less extensively explored. The present study contributes to this field by examining psychopathological symptoms in patients with ACD across different clinical presentations, extending the analysis beyond occupational exposure and hand‐limited disease.

Beyond the general comparison between the ACD and control groups, our analyses identified several sociodemographic factors associated with the distribution of psychopathological symptoms within the ACD group. In particular, age was positively associated with higher scores in phobic anxiety, psychoticism and the GSI. Although the cross‐sectional design does not allow causal inferences, these findings indicate that older age may be linked to a greater expression of specific psychopathological symptom dimensions in patients with ACD.

Women with ACD showed significantly higher scores than men across multiple psychopathological dimensions, including somatization, depression, anxiety, hostility, phobic anxiety, paranoid ideation and psychoticism. These findings are consistent with previous observations in chronic inflammatory skin diseases, where sex‐related differences in psychological symptom reporting have been described.

Regarding marital status, single patients exhibited higher scores in dimensions such as depression and psychoticism, while divorced or separated patients showed higher levels of paranoid ideation. Although causal relationships cannot be inferred, these associations may reflect the role of social and emotional context in the expression of psychopathological symptoms among patients with ACD.

Taken together, these results suggest that sex and social circumstances may be relevant factors to consider when assessing psychological symptoms in ACD, and support the value of a personalised and multidisciplinary approach in the clinical management of these patients.

As a chronic and recurrent condition, ACD may be accompanied by sustained psychological distress, which could be reflected in higher scores in certain psychopathological dimensions, including paranoid ideation. However, given the cross‐sectional design of the study, no causal inferences can be drawn regarding the direction of these associations. Educational level also emerged as a relevant factor associated with psychopathological symptom burden. Patients with a basic level of education showed higher scores in depression, GSI and PSDI compared with those with higher educational attainment. These differences may be related to disparities in health literacy, access to disease‐related information, or navigation of healthcare resources, although reverse causality, whereby chronic disease burden and psychological distress negatively impact educational and occupational trajectories, cannot be excluded. Taken together, these findings suggest that educational background should be considered when assessing psychological symptoms in patients with ACD and highlight the potential value of adapted educational and supportive strategies as part of a comprehensive management approach.

Previous studies have explored the relationship between disease severity in occupational contact dermatitis [26] and fragrance‐related dermatitis [27], and their impact on QoL. In line with this literature, our findings showed that ACD severity was positively associated with higher scores in interpersonal sensitivity, phobic anxiety and psychoticism. These associations indicate that greater clinical severity may be accompanied by higher levels of psychological symptoms expression, particularly in domains related to social functioning and emotional distress. However, given the cross‐sectional nature of the study, the direction of these relationships cannot be established. In addition, patients' self‐perceived disease severity was independently associated with higher levels of somatization and anxiety, underscoring the relevance of subjective illness perception in the expression of psychopathological symptoms among patients with ACD.

Emergency department visits among ACD patients were significantly associated with higher anxiety scores. This finding is consistent with previous reports indicating that individuals with higher levels of anxiety tend to make greater use of emergency healthcare services [28]. Such associations may reflect increased psychological distress in the context of disease flares or uncertainty regarding symptom management, although causal relationships cannot be inferred from the present data.

Conversely, no significant associations were observed between disease duration and the SCL‐90‐R dimensions, suggesting that chronicity alone may not fully account for the variability in psychopathological symptoms expression among the patients with ACD. Overall, these results point to an association between anxiety symptoms and patterns of emergency healthcare utilisation among patients with ACD.

Our findings support the view that ACD should be considered not only as a chronic inflammatory dermatosis with relevant physical manifestations, but also as a condition frequently accompanied by psychopathological symptoms. The observed associations warrant further research into the long‐term psychological correlates of ACD and their evolution over time. In this context, the results suggest that the systematic assessment of psychological symptoms may be a relevant component of a comprehensive clinical evaluation in patients with ACD. Future studies are needed to determine whether the integration of psychological assessment or supportive strategies into routine care may contribute to improved patient‐centred outcomes. This approach is supported by previous evidence from psychodermatology research in other chronic dermatological conditions, where the incorporation of psychological strategies has been associated with improvements in mental health‐related outcomes and treatment adherence [29].

Educational programmes, such as structured patient education programmes or patient schools, have been described as accessible strategies to support disease understanding and self‐management in chronic dermatological conditions, including aspects related to psychological well‐being [30]. In this context, such programmes may help patients develop coping strategies for common psychological symptoms, such as anxiety and stress, and promote greater engagement in self‐care behaviours.

In addition, psychological interventions, including cognitive‐behavioural therapy (CBT) and other mind–body approaches, have shown efficacy in reducing psychological distress and improving patient‐reported outcomes in other chronic dermatological diseases, such as psoriasis and atopic dermatitis. A recent Bayesian network meta‐analysis demonstrated that psychosocial interventions are effective in improving psychological outcomes in patients with psoriasis [31]. Similarly, integrative treatment approaches incorporating mind–body therapies have been proposed as beneficial adjunctive strategies in the management of atopic dermatitis, addressing symptoms such as anxiety, stress and itch‐related distress [32].

Although evidence specific to ACD remains limited, these findings suggest that psychological and psychosocial interventions effective in other chronic inflammatory skin diseases may represent promising avenues for future research and the development of integrated care models for patients with ACD and a high psychopathological symptom burden. Support groups and individualised psychological approaches may further contribute by addressing patients' emotional and social contexts, which are relevant dimensions in the experience of chronic skin disease. Further studies are needed to determine whether educational, psychological, or supportive interventions could influence patient‐reported outcomes or healthcare utilisation patterns in patients with ACD.

Complementing patch testing with the assessment of psychopathological symptoms within a multidisciplinary framework may contribute to a more comprehensive understanding of patients' needs in ACD. In situations where allergen avoidance alone does not fully address disease burden, psychological support may be considered as a complementary component of care, particularly in patients with a high psychological symptom burden [30]. These observations support the need for future research to further explore the potential role of psychological assessment and supportive interventions within holistic management approaches for ACD.

Psychopathological symptoms are increasingly recognised as a relevant component of dermatological disease. The field of psychodermatology describes the bidirectional interaction between skin disorders and mental health, whereby psychological symptoms may accompany, exacerbate, or interact with chronic cutaneous conditions. Previous reviews have identified depressive symptoms, anxiety, obsessive‐compulsive traits and psychotic symptoms as the most frequently encountered psychopathological manifestations in dermatological practice across a wide range of inflammatory and psychocutaneous diseases [33].

In recent years, growing attention has been directed toward psychodermatology as a structured and multidisciplinary field, with the development of liaison models and combined dermatology‐mental health clinics aimed at improving the recognition and management of psychocutaneous conditions [34]. Reviews of existing psychodermatology services suggest that integrated care models may help address unmet needs related to diagnostic complexity, fragmented care pathways and psychosocial comorbidity in patients with chronic skin diseases.

Beyond dermatological conditions, accumulating evidence indicates that mental health comorbidity and psychosocial factors are common features across a broad spectrum of skin and allergic diseases [35]. Large population‐based studies have shown that stress management ability, sociodemographic factors (including sex, age and educational level) and indicators of well‐being are significantly associated with the prevalence of common dermatological manifestations, supporting a biopsychosocial framework in skin disease [36].

Although ACD has been less extensively addressed within the broader allergic disease framework, accumulating evidence supports the relevance of considering mental health and psychosocial factors in this condition. Previous studies in patients with ACD have reported higher levels of perceived stress compared with controls [37]. These observations are consistent with the findings of the present study and reinforce the importance of taking psychological and psychosocial dimensions into account when evaluating patients with ACD.

This study has several limitations that should be acknowledged. First, its cross‐sectional and single‐centre design provides a snapshot of psychopathological symptoms at a single time point, limiting the ability to assess symptom trajectories, temporal relationships, or changes following allergen avoidance. Second, although all participants had a confirmed diagnosis of ACD, the heterogeneity of clinical presentations, such as lesion distribution or disease phenotypes, was not explored in depth and may have influenced the observed psychological profiles. These aspects warrant further investigation in future studies.

In addition, psychopathological symptoms were assessed using self‐reported measures, which may be influenced by reporting bias. Although SCL‐90‐R subscale and global index scores represent continuous composite measures, some score distributions showed deviations from normality, which may affect the precision of parametric statistical modelling. To address this issue, sensitivity analyses using non‐parametric methods were performed, and results were interpreted cautiously. Finally, the control group was not formally screened for subclinical psychological symptoms, which may have led to an underestimation of psychological distress in the comparison group.

Taken together, these limitations underscore the need for multicentre, longitudinal studies incorporating refined clinical phenotyping and alternative analytical approaches to further validate and extend the present findings.

In conclusion, patients with ACD experience higher levels of psychopathological symptoms compared with controls. These findings highlight the relevance of the psychological dimension of ACD and support its consideration as part of a comprehensive clinical evaluation. Further research is warranted to clarify the potential role of psychological assessment and supportive strategies within integrated care approaches for patients with ACD.

## Author Contributions

**Francisco José Navarro‐Triviño:** conceptualization, investigation, writing – original draft, methodology, validation, visualization, writing – review and editing, formal analysis, project administration, data curation. **Ricardo Ruiz‐Villaverde:** writing – original draft, writing – review and editing, validation. **Álvaro Prados‐Carmona:** writing – original draft, writing – review and editing, validation. **María Isabel Peralta Ramirez:** writing – original draft, writing – review and editing, validation.

## Funding

‐ Funding for open access charge: Universidad de Granada / CBUA

## Conflicts of Interest

The authors declare no conflicts of interest.

## Data Availability

The data that support the findings of this study are available from the corresponding author upon reasonable request.

## References

[1] T. U. R. H. C. Edwards , “Allergic Contact Dermatitis,” Cutis 112 (2023): 195–197. 37988316 10.12788/cutis.0868

[2] F. Alinaghi , N. H. Bennike , A. Egeberg , J. P. Thyssen , and J. D. Johansen , “Prevalence of Contact Allergy in the General Population: A Systematic Review and Meta‐Analysis,” Contact Dermatitis 80 (2019): 77–85. 30370565 10.1111/cod.13119

[3] D. Bonamonte , C. Foti , M. Vestita , and G. Angelini , “Noneczematous Contact Dermatitis,” ISRN Allergy 2013 (2013): 1–10. 10.1155/2013/361746 PMC3787648 24109520

[4] P. Korkoliakou , V. Efstathiou , I. Giannopoulou , et al., “Psychopathology and Alexithymia in Patients With Psoriasis,” Anais Brasileiros de Dermatologia 92 (2017): 510–515. 28954100 10.1590/abd1806-4841.20175660 PMC5595598

[5] K. Koszorú , J. Borza , L. Gulácsi , and M. Sárdy , “Quality of Life in Patients With Atopic Dermatitis,” Cutis 104 (2019): 174–177. 31675393

[6] H. Baurecht , C. Welker , S.‐E. Baumeister , et al., “Relationship Between Atopic Dermatitis, Depression and Anxiety: A Two‐Sample Mendelian Randomization Study,” British Journal of Dermatology 185 (2021): 781–786. 33817779 10.1111/bjd.20092

[7] M. Esposito , A. Giunta , R. C. Nanni , et al., “Depressive Symptoms and Insecure Attachment Predict Disability and Quality of Life in Psoriasis Independently From Disease Severity,” Archives of Dermatological Research 313 (2021): 431–437. 32776228 10.1007/s00403-020-02116-8 PMC8238751

[8] N. Salari , P. Heidarian , A. Hosseinian‐Far , F. Babajani , and M. Mohammadi , “Global Prevalence of Anxiety, Depression, and Stress Among Patients With Skin Diseases: A Systematic Review and Meta‐Analysis,” Journal of Prevention 45 (2024): 611–649. 38822990 10.1007/s10935-024-00784-0

[9] M. Siewertsen , C. Näslund‐Koch , J. Duus Johansen , et al., “Psychological Burden, Anxiety, Depression and Quality of Life in Patients With Hand Eczema: A Systematic Review and Meta‐Analysis,” Journal of the European Academy of Dermatology and Venereology 38 (2024): 2110–2117. 38808968 10.1111/jdv.20140

[10] M. Iannone , A. Janowska , S. Panduri , et al., “Impact of Psychiatric Comorbidities in Psoriasis, Hidradenitis Suppurativa and Atopic Dermatitis: The Importance of a Psychodermatological Approach,” Experimental Dermatology 31 (2022): 956–961. 35285091 10.1111/exd.14563 PMC9314578

[11] P. N. Woo , I. C. Hay , and A. D. Ormerod , “An Audit of the Value of Patch Testing and Its Effect on Quality of Life,” Contact Dermatitis 48 (2003): 244–247. 12868963 10.1034/j.1600-0536.2003.00113.x

[12] M. Koskelo , S. Sinikumpu , J. Jokelainen , and L. Huilaja , “Anxiety and Depression in Patients With Hand Eczema: A Population‐Based Study Among 853 Middle‐Aged Subjects,” Contact Dermatitis 89 (2023): 464–470. 37647940 10.1111/cod.14412

[13] J. D. Johansen , K. Aalto‐Korte , T. Agner , et al., “European Society of Contact Dermatitis Guideline for Diagnostic Patch Testing – Recommendations on Best Practice,” Contact Dermatitis 73 (2015): 195–221. 26179009 10.1111/cod.12432

[14] E. Simpson , R. Bissonnette , L. F. Eichenfield , et al., “The Validated Investigator Global Assessment for Atopic Dermatitis (vIGA‐AD): The Development and Reliability Testing of a Novel Clinical Outcome Measurement Instrument for the Severity of Atopic Dermatitis,” Journal of the American Academy of Dermatology 83 (2020): 839–846. 32344071 10.1016/j.jaad.2020.04.104

[15] R. Bhatia , V. K. Sharma , M. Ramam , G. Sethuraman , and C. P. Yadav , “Clinical Profile and Quality of Life of Patients With Occupational Contact Dermatitis From New Delhi, India,” Contact Dermatitis 73 (2015): 172–181. 25990826 10.1111/cod.12411

[16] Y. Benyamini , D. Goner‐Shilo , and A. Lazarov , “Illness Perception and Quality of Life in Patients With Contact Dermatitis,” Contact Dermatitis 67 (2012): 193–199. 22612452 10.1111/j.1600-0536.2012.02071.x

[17] L. R. Derogatis , SCL‐90‐R: Symptom Checklist‐90‐R. Administration, Scoring and Procedures Manual (NCS Pearson, 1994).

[18] J. I. Robles Sánchez , J. Manuel , A. Rodríguez , et al., “SCL‐90‐R: Application and Analysis of Its Psychometric Properties in a Sample of Spanish Clinical Subjects,” in Clinical, Legal and Forensic Psychopathology, vol. 2 (Universidad Complutense de Madrid, 2002), 1–19.

[19] S. Tsiori , N. Rompoti , K. Kontoangelos , C. Papageorgiou , A. Stratigos , and D. Rigopoulos , “Psychopathology and Alexithymia in Patients With Moderate‐To‐Severe Psoriasis: Development of a Novel Index With Prognostic Value,” International Journal of Environmental Research and Public Health 19 (2022): 4029. 35409713 10.3390/ijerph19074029 PMC8998217

[20] P. Staubach , M. Dechene , M. Metz , et al., “High Prevalence of Mental Disorders and Emotional Distress in Patients With Chronic Spontaneous Urticaria,” Acta Dermato‐Venereologica 91 (2011): 557–561. 21597672 10.2340/00015555-1109

[21] N. Konuk , R. Koca , L. Atik , S. Muhtar , N. Atasoy , and B. Bostanci , “Psychopathology, Depression and Dissociative Experiences in Patients With Lichen Simplex Chronicus,” General Hospital Psychiatry 29 (2007): 232–235. 17484940 10.1016/j.genhosppsych.2007.01.006

[22] T. Agner , K. E. Andersen , F. M. Brandao , et al., “Hand Eczema Severity and Quality of Life: A Cross‐Sectional, Multicentre Study of Hand Eczema Patients,” Contact Dermatitis 59 (2008): 43–47. 18537992 10.1111/j.1600-0536.2008.01362.x

[23] R. S. Cvetkovski , R. Zachariae , H. Jensen , J. Olsen , J. D. Johansen , and T. Agner , “Quality of Life and Depression in a Population of Occupational Hand Eczema Patients,” Contact Dermatitis 54 (2006): 106–111. 16487283 10.1111/j.0105-1873.2006.00783.x

[24] A. Kouris , K. Armyra , C. Christodoulou , et al., “Quality of Life, Anxiety, Depression and Obsessive‐Compulsive Tendencies in Patients With Chronic Hand Eczema,” Contact Dermatitis 72 (2015): 367–370. 25693684 10.1111/cod.12366

[25] N. Pondeljak , T. Lucija , L. M. Elvira , Š. Mirna , K. Dalibor , and L. M. Liborija , “Salivary Cortisol and Perceived Psychological Stress in Patients With Chronic Contact Hand Dermatitis,” Contact Dermatitis 89 (2023): 393–395. 37579769 10.1111/cod.14399

[26] M. Y. Z. Lau , M. C. Matheson , J. A. Burgess , S. C. Dharmage , and R. Nixon , “Disease Severity and Quality of Life in a Follow‐Up Study of Patients With Occupational Contact Dermatitis,” Contact Dermatitis 65 (2011): 138–145. 21722138 10.1111/j.1600-0536.2011.01896.x

[27] N. H. Bennike , M. S. Heisterberg , I. R. White , et al., “Quality of Life and Disease Severity in Dermatitis Patients With Fragrance Allergy‐A Cross‐Sectional European Questionnaire Study,” Contact Dermatitis 81 (2019): 89–96. 30802323 10.1111/cod.13252

[28] B. Abar , A. Holub , J. Lee , V. DeRienzo , and F. Nobay , “Depression and Anxiety Among Emergency Department Patients: Utilization and Barriers to Care,” Academic Emergency Medicine 24 (2017): 1286–1289. 28741875 10.1111/acem.13261

[29] T. Samela , G. Cordella , V. Antinone , P. Sarandrea , A. R. Giampetruzzi , and D. Abeni , “The Use of SCL‐K‐9 to Measure General Psychopathology in Women and Men With Skin Conditions,” Frontiers in Psychology 13 (2022): 977264, 10.3389/fpsyg.2022.977264. 36337481 PMC9632958

[30] C. J. Connor , “Management of the Psychological Comorbidities of Dermatological Conditions: Practitioners' Guidelines,” Clinical, Cosmetic and Investigational Dermatology 10 (2017): 117–132. 28458571 10.2147/CCID.S111041 PMC5404497

[31] L. Lu , Y. Xu , M. Shi , and A. Liu , “Psychosocial Interventions for Psoriasis: A Bayesian Network Meta‐Analysis,” Journal of Dermatological Treatment 36 (2025): 2427321, 10.1080/09546634.2024.2427321. 40331790

[32] G. Yosipovitch , L. Canchy , B. R. Ferreira , et al., “Integrative Treatment Approaches With Mind–Body Therapies in the Management of Atopic Dermatitis,” Journal of Clinical Medicine 13 (2024): 5368. 39336855 10.3390/jcm13185368 PMC11432615

[33] S. M. Rahman , A. Abduelmula , and M. Jafferany , “Psychopathological Symptoms in Dermatology: A Basic Approach Toward Psychocutaneous Disorders,” International Journal of Dermatology 62 (2023): 346–356. 35816285 10.1111/ijd.16344

[34] A. Patel and M. Jafferany , “Multidisciplinary and Holistic Models of Care for Patients With Dermatologic Disease and Psychosocial Comorbidity,” JAMA Dermatology 156 (2020): 686. 32347896 10.1001/jamadermatol.2020.0394

[35] A. E. Conway , M. Verdi , N. Kartha , et al., “Allergic Diseases and Mental Health,” Journal of Allergy and Clinical Immunology. In Practice 12 (2024): 2298–2309. 38851487 10.1016/j.jaip.2024.05.049 PMC13728810

[36] A. Kubrak , A. Zimny‐Zając , S. Makuch , et al., “Psychosocial Factors, Stress, and Well‐Being: Associations With Common Dermatological Manifestations in a Large Polish Cross‐Sectional Analysis,” Journal of Clinical Medicine 14 (2025): 3943. 40507703 10.3390/jcm14113943 PMC12156280

[37] F. J. Navarro‐Triviño , Á. Prados‐Carmona , R. Ruiz‐Villaverde , and M. I. Peralta‐Ramírez , “Impact of Perceived Stress, Locus of Control, and Self‐Efficacy on Allergic Contact Dermatitis,” Healthcare 13 (2025): 2498. 41095584 10.3390/healthcare13192498 PMC12524717

